# Identification of Genes Involved in the Glycosylation of Modified Viosamine of Flagellins in *Pseudomonas syringae* by Mass Spectrometry

**DOI:** 10.3390/genes2040788

**Published:** 2011-10-28

**Authors:** Masanobu Yamamoto, Mayumi Ohnishi-Kameyama, Chi L. Nguyen, Fumiko Taguchi, Kazuhiro Chiku, Tadashi Ishii, Hiroshi Ono, Mitsuru Yoshida, Yuki Ichinose

**Affiliations:** 1 National Food Research Institute, 2-1-12 Kannondai, Tsukuba, Ibaraki 305-8642, Japan; E-Mails: ymasanob@affrc.go.jp (M.Y.); kameyama@affrc.go.jp (M.O.-K.); kchiku@affrc.go.jp (K.C.); tishii@affrc.go.jp (T.I.); ono@affrc.go.jp (H.O.); mitsuru@affrc.go.jp (M.Y.); 2 The Graduate School of Natural Science and Technology, Okayama University, Tsushima-naka 1-1-1, Okayama 700-8530, Japan; E-Mails: linhchiagi@yahoo.com (C.L.N.); ftaguchi@cc.okayama-u.ac.jp (F.T.)

**Keywords:** flagellin, glycosylation, mass spectrometry, viosamine island

## Abstract

Previously we revealed that flagellin proteins in *Pseudomonas syringae* pv. *tabaci* 6605 (Pta 6605) were glycosylated with a trisaccharide, modified viosamine (mVio)-rhamnose-rhamnose and that glycosylation was required for virulence. We further identified some glycosylation-related genes, including *vioA, vioB, vioT, fgt1*, and *fgt2*. In this study, we newly identified *vioR* and *vioM* in a so-called viosamine island as biosynthetic genes for glycosylation of mVio in Pta 6605 by the mass spectrometry (MS) of flagellin glycan in the respective mutants. Furthermore, characterization of the mVio-related genes and MS analyses of flagellin glycans in other pathovars of *P. syringae* revealed that mVio-related genes were essential for mVio biosynthesis in flagellin glycans, and that *P. syringae* pv. *syringae* B728a, which does not possess a viosamine island, has a different structure of glycan in its flagellin protein.

## 1. Introduction

Protein glycosylation is found not only in eukaryotes but also in prokaryotes. Although bacterial glycoproteins were found in various animal pathogenic bacteria, including *Pseudomonas aeruginosa, Campylobacter coli, C. jejuni, Helicobacter pylori, Aeromonas caviae, Escherichia coli*, and *Neisseria meningitides*, the reports of glycoproteins in phytopathogenic bacteria were restricted to *P. syringae* and *Acidovorax avenae* [1–4]. Most bacterial glycoproteins were found in the surface proteins of the organism, such as pilins, flagellins, and S-layer proteins. Glycosylation of flagellins was reported to be important for virulence in animal pathogenic bacteria such as *P. aeruginosa* and *C. jejuni* [5,6] and in plant pathogenic bacteria *P. syringae* pv. *tabaci* (Pta) 6605 and pv. *glycinea* (Pgl) race 4 [7,8]. We previously reported that flagellin glycans from Pta 6605 and Pgl race 4 were composed of two or three rhamnosyl residues and one d-Qui*p*4*N*(3-hydroxy-1-oxobutyl)2Me residue (modified viosamine, mVio) [9–12]. We further determined the distal end of the flagellin glycan to be mVio and the genetic region required for the synthesis of mVio in Pta 6605 [11]. There are seven open reading frames (ORFs), including *vioA, vioB*, and *vioT*, between the conserved glutamine synthase gene (gs) and the histidine kinase gene (*hk*) in Pta 6605 [11]. We designated the region as a viosamine island. However, the function of other gene products remained to be elucidated.

In this study, we newly generated defective mutant strains for putative genes for methyltransferase and 3-oxoacyl-(acyl-carrier-protein) reductase, and we investigated the molecular masses of flagellin proteins and their glycopeptides. The results of homology searches suggested a metabolic pathway that produces thymidine diphosphate (dTDP)-*N*-(3-hydroxy-1-oxobutyl)-2-*O*-methylviosamine (dTDP-mVio) and the functions of the products of seven ORFs in the viosamine island. Furthermore, we expand the analysis of flagellin glycan in *P. syringae* pv. *phaseolicola* (Pph) 1448A, pv. *tomato* (Pto) DC3000, and pv. *syringae* (Psy) B728a, in which whole genomic sequences were already published [13–15]. We determined molecular masses of flagellin glycan of each strain and compared the organization of various viosamine islands. Recent draft sequences of genomic DNA of pv. *glycinea* race 4 and str. B076 [16], pv. *savastanoi* NCPPB 3335 [17], pv. *oryzae* 1_6 [18], pv. *tabaci* 11528 [19] and 6605 (Dr. Studholme, personal communication), pv. *tomato* T1 [20], NCPPB1108 [21] and Max13 [22] and pv. *syringae* 642 [23], and pv. *aesculi* 2250 and NCPPB 3681 [24] and pv. *syringae* FF5 [22] enable us to compare the genomic organization of various viosamine islands. The classification of a viosamine island consists of intrapathovar variation in *P. syringae*. Structural analyses of genomic organization and biochemical analyses of flagellin glycans revealed that flagellin glycans have both universality and diversity.

## 2. Results and Discussion

### 2.1. Comparison of the Viosamine Island in Pta 6605 and the Glycosylation Island in P. aeruginosa PAK Strain and the Generation of vioM and vioR Mutant Strains

**Figure 1:**
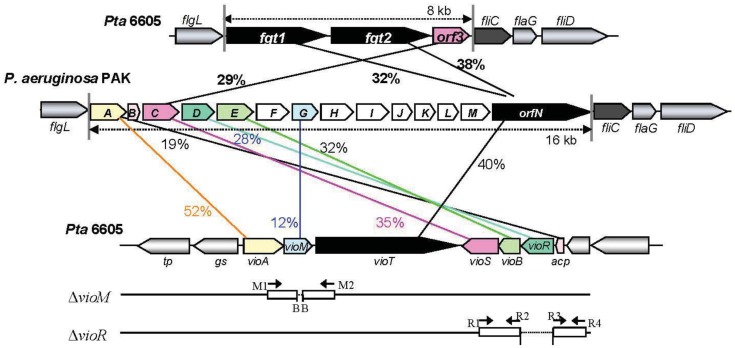
Viosamine island in Pta 6605 and generation of *vioM* and *vioR* mutant strains. The nucleotide sequences for the glycosylation and viosamine islands of Pta 6605 and *P. aeruginoas* PAK were deposited in the DDBJ, EMBL and GenBank Nucleotide Sequence Databases under the accession number AB499894, AB061230 and AF332547, respectively. Each gene is named by the putative function of the product: transporter (*tp*), glutamine synthetase (gs), dTDP-viosamine aminotransferase (*vioA*), methyltransferase in modified viosamine (mVio) (*vioM*), mVio transferase (*vioT*), 3-oxoacyl-(acyl-carrier-protein) synthase III (*vioS*), dTDP-viosamine acetyltransferase (*vioB*), 3-oxoacyl-(acyl-carrier-protein) reductase (*vioR*), and acyl-carrier protein (*acp*). Primers used for PCR are indicated by arrows. Each primer sequence is described in the **Experimental Section**. The flagellar gene cluster from *flgL* to *fliD* in Pta 6605 and the *P. aeruginosa* PAK strain is also shown with a comparison of homologous genes. Percentages show amino acid identity.

A viosamine island was previously isolated from Pta 6605 by PCR methods [11]. A detailed homology search of seven ORFs in this island revealed that all ORFs had corresponding homologous genes in a glycosylation island in the *P. aeruginosa* PAK strain [25] as shown in Figure 1. These results indicate that both flagellins from Pta 6605 and *P. aeruginosa* PAK strain may possess similar glycan. Major components of flagellin glycan were reported to be rhamnose, mannose, glucose and 4-amino-4,6-dideoxyglucose (viosamine) in *P. aeruginosa* PAK strain, although its whole structure was not elucidated yet [26]. It is not known whether each gene in viosamine cluster of *P. aeruginosa* has the same function to the corresponding homologous gene, because each amino acid identity was relatively low. However, the genes in viosamine cluster in *P. aeruginosa* seem to also contribute the modification of flagellin glycan. We found that not only the *vioA, vioB*, and *vioT* genes but also four other genes may be involved in the glycosylation of flagellin by the synthesis of dTDP-mVio. Therefore, we redesignated the gene homologous to methyltransferase as *vioM*, 3-oxoacyl-(acyl-carrier protein) synthase III as *vioS*, 3-oxoacyl-(acyl-carrier protein) reductase as *vioR*, and *hp2* (hypothetical protein gene 2) as *acp*. Similarities of the deduced amino acid sequences of VioM *vs.* OrfG, VioS vs. OrfC, VioR *vs.* OrfD, and ACP *vs.* OrfB are 12%, 35%, 28%, and 19%, respectively (Table 1). Sequence-specific deletion of *vioM* or *vioR* was confirmed by genomic PCR and sequencing.

**Table 1:** Similarities of open reading frames (ORFs) in viosamine cluster in Pta 6605 to those in *P. aeruginosa* (Pa) PAK strain.

**Gene in Pta 6605** *	**Size of product (amino acid)**	**Putative function of gene product**	**Homologous gene in Pa PAK**	**% Identity (amino acid)**
*vioA* (*vioA*)	372	dTDP-viosamine aminotransferase	*orfA*	52%
*vioM* (*mt*)	241	methyltransferase	*orfG*	12%
*vioT* (*vioT*)	1,173	modified viosamine transferase	*orfN*	40%
*vioS* (*3o-acpS3*)	338	3-oxoacyl-(acyl-carrier protein) synthase III	*orfC*	35%
*vioB* (*vioB*)	213	dTDP-viosamine acetyltransferase	*orfE*	32%
*vioR* (*3o-acpR*)	253	3-oxoacyl-(acyl-carrier protein) reductase	*oefD*	28%
*acp* (*hp2*)	69	acyl-carrier protein	*orfB*	19%

* Gene names reported in Nguyen *et al.* (2009) were indicated in the parentheses.

### 2.2. Effects of Each Mutation of vioM and vioR on Flagellin Glycosylation in Pta 6605

**Figure 2:**
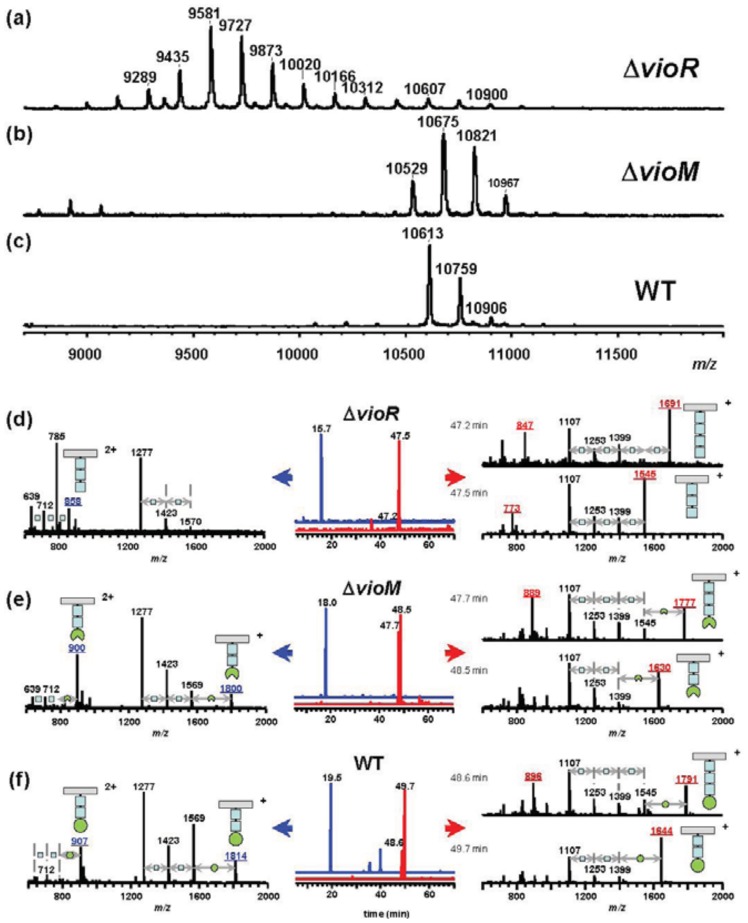
Mass spectra of digested flagellins of **(a,d)** Δ*vioR;* **(b,e)** Δ*vioM;* and **(c,f)** the WT of Pta 6605. Upper spectra (**a–c**) were of trypsin-digested flagellins by MALDI-TOF MS. The average masses of the peptide N136-R210 and the predominant trisaccharide glycan for the WT, mVio-Rha-Rha, are 7,387 and 555.5, respectively. The mVio-lacking glycan is composed of rhamnose whose residue has a mass of 146 in glycan chains. The mutant lacking *vioR* showed many regularly spaced peaks at the head of the list at *m/z* 9,581 (≈7,387 + (146) × 15 + 1). The protonated ions of the glycopeptides including six glycans were observed at *m/z* 10,613 (7,387 + (537.5) × 6 + 1) for the WT and *m/z* 10,529 (7,387 + (523.5) × 6 + 1) for Δ*vioM* strains. The accompanied ions with 146 and 292 larger mass values are of glycopeptides with one and two tetrasaccharide glycans out of six glycans, respectively. The lower spectra (**d**–**f**) were of Asp-*N*-digested flagellins by LC-ESI/MS. The middle column shows extracted ion chromatograms of *m/z* 1,277 (in red) and 1,107 (in blue) corresponding to the mass values of amino acid sequences D200-A211 and E189-I199, respectively. In the mass spectra (left and right columns), underlined mass values are of [M+H]^+^ and [M+2H]^2+^ of molecular-related ions, and the graphical assignments are as follows: grey rectangle: peptide; blue square: Rha; green circle: mVio; and notched circle: demethylated mVio. The symbol “+” or “2+” means the ion is singly charged or doubly charged, respectively.

To assess the effects of gene deletion, we analyzed flagellins in Δ*vioR* and Δ*vioM* mutants by mass spectrometry. Glycosylated amino acids in Pta 6605 flagellin were previously identified at Ser143, Ser164, Ser176, Ser183, Ser193, and Ser201 residues [8]. We digested each flagellin by trypsin to produce a glycopeptide, N136-R210, that included all six glycosylation sites to detect the mass differences among mutants by matrix-assisted laser desorption/ionization—time of flight (MALDI-TOF) mass spectrometry. The MALDI-TOF mass spectrum of Δ*vioR* flagellin showed a polymer-like pattern over a wide *m/z* range (9,000–11,000) with a regular peak-to-peak distance of 146 (Figure 2(a)). This is a characteristic profile in glycopeptides with a homoglycan, as we previously reported for Δ*vioA*, Δ*vioB*, or Δ*vioT* flagellins whose glycans were composed of rhamnoses [11]. The distribution pattern of the most intense peak at *m/z* 9,580 suggested that out of six glycans of flagellin, three glycans were trisaccharides and the other glycans were disaccharides.

Smaller glycopeptide fragments including one or two glycans were obtained by Asp-*N* digestion of flagellin, and those of Δ*vioR* flagellin were analyzed by liquid chromatography (LC)-electrospray ionization (ESI) mass spectrometry (MS) (Figure 2(d)). In the extracted ion chromatogram (XIC), the peptide D200-A211 including a glycan was observed at 15.7 min (Figure 2(d), middle, in blue), which was almost the same retention time as those of Δ*vioA*, Δ*vioB*, or Δ*vioT* flagellins (data not shown). The mass spectrum of the peak at 15.7 min gave doubly charged molecular ion peaks at *m/z* 858 and *m/z* 785 (Figure 2(d), left), indicating that the glycan at Ser201 was made up of two or three deoxyhexoses. E189-I199, another glycopeptide including a glycan, gave two peaks in the XIC (Figure 2(d), middle, in red). The mass spectra indicated the glycan was composed of three or four deoxyhexoses (Figure 2(d), right). The other glycopeptides containing two glycans, D139-F167 and D168-A188, afforded similar LC-ESI/MS data to those of Δ*vioA*, Δ*vioB*, or Δ*vioT* flagellins (data not shown), suggesting that Δ*vioR* flagellin was almost the same as those mutants. Accordingly, the Δ*vioR* mutant was clarified to contain rhamnosyl glycans without modified viosamines for the flagellin.

Meanwhile, the Δ*vioM* mutant gave ion peaks for glycopeptide N136-R210 at *m/z* 10,529, 10,675, and 10,821 in the MALDI-TOF mass spectrum (Figure 2(b)) similar to those of the wild type (WT) flagellin (Figure 2(c)) rather than those of Δ*vioR* (Figure 2(a)). The *m/z* values of the Δ*vioM* mutant were 84 smaller than the corresponding values of the WT, 10,613, 10,759, and 10,906, respectively. We compared the results from LC-ESI/MS analyses of Asp-*N*-digested glycopeptides of the Δ*vioM* mutant and the WT flagellin (Figures 2(e,f), respectively). The peptides D200-A211 and E189-I199 of Δ*vioM* were eluted at 18.0 min and 47.7 - 48.5 min, respectively (Figure 2(e), middle). The protonated molecular ion ([M+H]^+^) of D200-A211 was observed at *m/z* 1,800, accompanied by the deglycosylated fragment ions at *m/z* 1,569 ([M+H−231]^+^), 1,423 ([M+H−231−146]^+^), and 1,277 ([M+H−231−146−146]^+^) (Figure 2(e), left). The glycopeptide E189-I199 at 47.7 min and 48.5 min showed [M+H]^+^ at *m/z* 1,777 and *m/z* 1,630, respectively (Figure 2(e), right). The accompanying deglycosylated fragment ions corresponded to [M+H−231]+ and [M+H−231−(146)_n_]+ (Figure 2(e), right). Out of these ions, only the [M+H]^+^ ions gave 14 different *m/z* values from those of the WT (Figure 2(f), left and right). This meant the terminal saccharide of the glycan of Δ*vioM* differed from that of the WT. The 14 smaller *m/z* values and the slightly earlier retention times of Δ*vioM* glycopeptides compared to those of the WT (Figure 2(f)) indicated that each glycan of Δ*vioM* included mVio without a methyl group. The deletion of *vioM* resulted in the production of demethylated mVio at the non-reducing end of the glycan.

### 2.3. Putative Biosynthetic Pathway of dTDP-mVio

**Figure 3:**
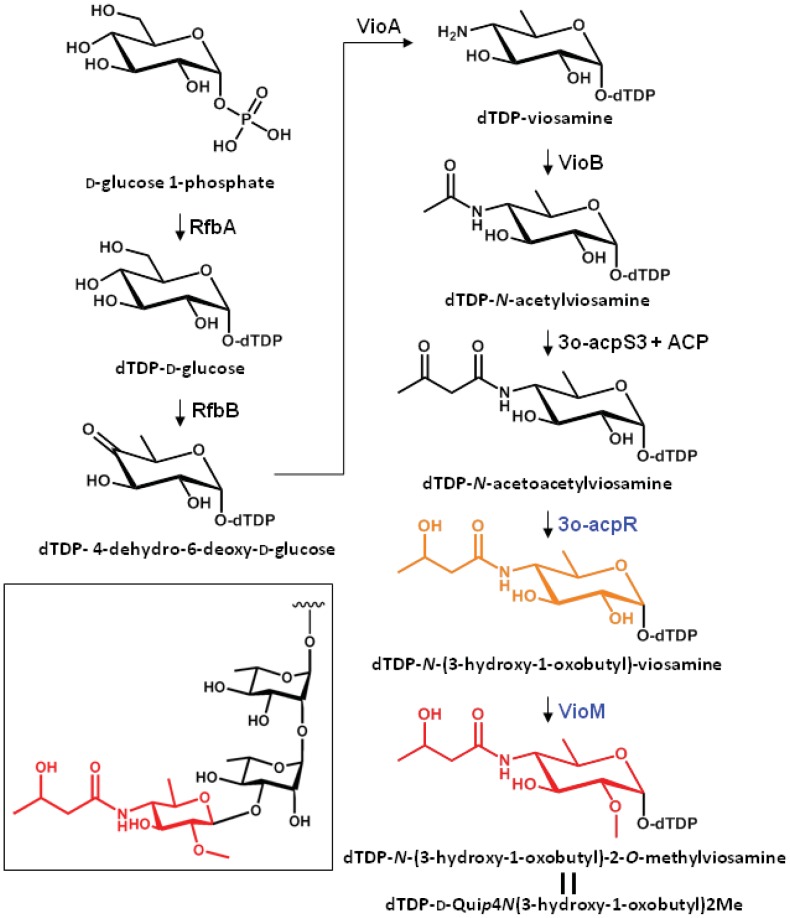
The metabolic pathway to produce modified viosamine and the structure of glycan attached to S201 in the flagellin (inset). Only the modified viosamine (in red) and the last precursor (in orange) were transferred to the non-reducing end of the rhamnosyl glycan by vioT.

The putative biosynthetic pathway of dTDP-mVio is illustrated in Figure 3. In a previous study we clarified that mVio was not transferred to the rhamnosyl glycan of flagellin in the mutants lacking *vioA, vioB*, and *vioT* [11]. Additionally, we revealed that the flagellin glycans in the *vioR* mutant were also composed of only rhamnose. On the other hand, the *vioM* mutant had demethylated mVio at the non-reducing end of flagellin glycans. These results indicated that the mVio-transfer enzyme, VioT, strictly recognized the modification of the amino group at position 4 of viosamine, and dTDP-*N*-acetoacetylviosamine and its precursors were not suitable for VioT as substrates. As a result, VioT recognizes dTDP-*N*-(3-hydroxy-1-oxobutyl)-2-*O*-methylviosamine and dTDP-*N*-(3-hydroxy-1-oxobutyl)-viosamine as the substrates and transfers *N*-(3-hydroxy-1-oxobutyl)-2-*O*-methylviosamine and *N*-(3-hydroxy-1-oxobutyl)-viosamine to the non-reducing terminus of rhamnosyl glycans.

### 2.4. Comparison of the Viosamine Island among Different Pathovars of P. syringae

**Figure 4:**
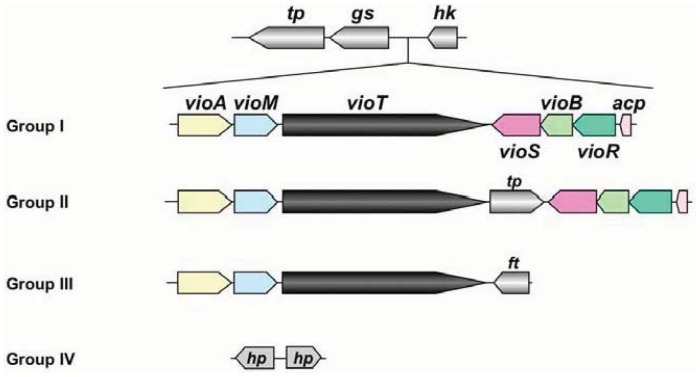
Gene cluster in the viosamine island in several pathovars of *P. syringae*. The genomic regions corresponding to viosamine islands from pathovars and strains from *P. syringae* were classified into four groups. Group I contains those from pv. *tabaci* 6605, pv. *tabaci* ATCC 11528, pv. *glycinea* race 4, pv. *glycinea* B076, pv. *savastanoi* NCPPP 3335, pv. *phaseolicola* 1148A, and pv. *aesculi* 2250 and NCPPB 3681; Group II contains those from pv. *tomato* DC3000, T1, Max 13, and NCPPB 1108; Group III contains those from pv. *syringae* 642; and Group IV contains those from pv. *syringae* B728a and FF5 and pv. *oryzae* 1_6. The following abbreviations are used: *tp:* major facilitator family transporter; *ft:* folate-dependent phosphoribosylglycinamide formyltransferase PurN; *hp:* hypothetical protein. Other gene names are indicated in the legend of Figure 1.

At present, genomic sequences from 8 pathovars and altogether 16 strains have been determined in *P. syringae* [16]. The genomic information of these pathogens revealed that the organization of the viosamine island can be divided into four groups, as shown in Figure 4. Pathovar *tabaci* strains 6605 and ATCC 11528, pv. *glycinea* strains race 4 and B076, pv. *aesculi* strains 2250 and NCPPB 3681, pv. *savastanoi* NCPPP 3335 and pv. *phaseolicola* 1148A (Pph 1448A) belong to Group I; all strains of pv. *tomato* including DC3000 (Pto DC3000) are Group II; pv. *syringae* strain 642 belongs to Group III; and pv. *oryzae* 1_6 and pv. *syringae* strains B728a (Psy B728a) and FF5 were Group IV. Of these strains, the majority belong to Groups I and II, and these strains conserve all ORFs in Pta 6605. This genomic organization suggested that all strains belonging to Groups I and II produce the same mVio-Rha-Rha glycans in their flagellins. However the Group III strain does not possess *vioS, vioB, vioR*, or *acp*. We demonstrate that VioT in Pta 6605 does not transfer dTDP-viosamine, because the Δ*vioB* mutant does not possess viosamine-related saccharides in the flagellin glycan [11]. There are no homologs of *vioS, vioB, vioR* and *acp* in Group III pv. *syringae* 642. Therefore, it is doubtful that VioT in pv. *syringae* 642 is functional. Although there are two genes in this region in the Group IV strains, these genes presumably encode hypothetical proteins and do not show homology to any genes in this region in other pathovars/strains of *P. syringae*. We found that mVio is required to attenuate the elongation of rhamnoses [11]. However, there seem to be no viosamine-related saccharides in Groups III and IV of *P. syringae*, suggesting that other saccharides may alternate attenuation of flagellin glycan elongation.

Phylogenetic analysis of *P. syringae* pathovars using seven housekeeping genes showed that the pathovars can be classified into five clades [27]. All pathovars/strains classified into clade 3 belong to Group I in the classification of the viosamine island, and clade 1 belongs to Group II. Furthermore, clade 2c including pv. *syringae* 642 belongs to Group III, and clade 2b including pv. *syringae* strains B728a and FF5, and clade 4 including pv. *oryzae* 1_6 belongs to Group IV. Thus, the classification of the pathovars based on the viosamine island is fairly consistent with the classification by the housekeeping genes.

### 2.5. Analysis of Flagellin Glycan from Pph 1448A, Pto DC3000, and Psy B728a

**Figure 5:**
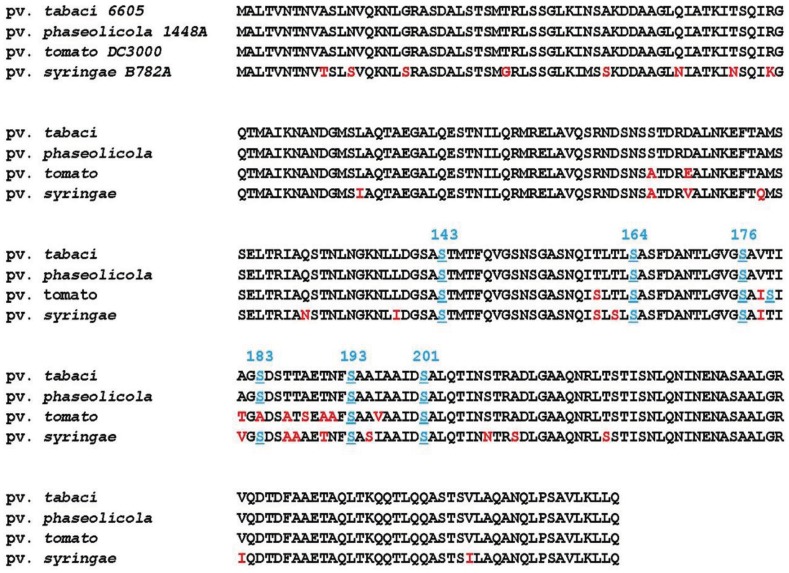
Comparison of amino acid sequences of flagellins among pathovars in *P. syringae*. Underlined light blue serine residues are glycosylated. Red letters in the sequences of pvs. *tomato* DC3000 and *syringae* B728A show different amino acids from that of pv. *tabaci* 6605.

**Figure 6:**
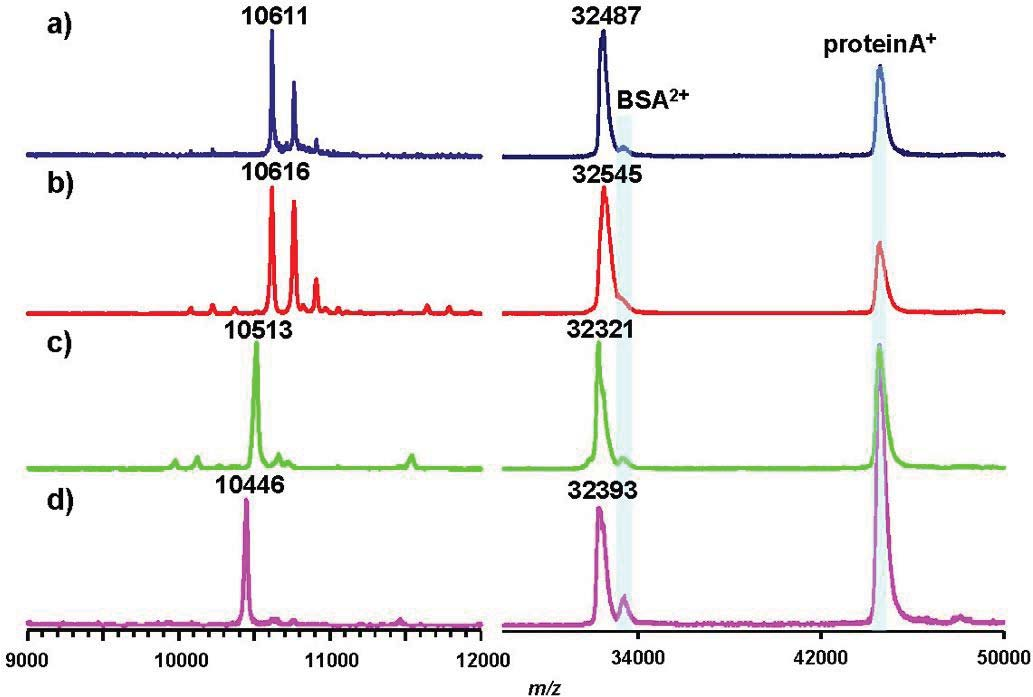
Comparison of MALDI-TOF mass spectra among pathovars in *P. syringae*. Mass spectra on the left side are of trypsin-digested flagellin polypeptide (N136-R210) of pv. *tabaci* 6605 (**a**), pv. *phaseolicola* 1448A (**b**), pv. *tomato* DC3000 (**c**), and pv. *syringae* B728a (**d**). Those on the right side are of intact flagellins. Blue peaks are of the calibration standard mixture.

As representative pathovars/strains of Groups I, II, and IV, we investigated flagellin glycans of Pph 1448A (Group I), Pto DC3000 (Group II), and Psy 728a (Group IV). The amino acid sequence of flagellin from Pph 1448A was identical to that from Pta 6605, and the amino acid identities of Pta flagellin to pv. *tomato* DC3000 and pv. *syringae* B728a were 96% and 90%, respectively (Figure 5). MALDI-TOF mass spectra of whole flagellin proteins of Pph 1448A, Pto DC3000, and Psy B728a are shown in Figure 6 (right). All flagellins gave [M+H]^+^ at around *m/z* 32,000. The differences between calculated and observed *m/z* values were due to the existence of glycans (Table 2), and were approximately 3,200∼3,400. The mass spectra of trypsin-digested glycopeptides N136-R210, including six glycosylation sites, are shown in Figure 6 (left). The results were in good agreement with those of intact proteins, indicating all glycans of flagellins were linked to the glycopeptides N136-R210.

Pph 1448A (Group I) flagellin had the same amino acid sequence as that of Pta 6605, and gave a similar *m/z* value of [M+H]^+^ with Pta 6605, indicating that the flagellin glycan in Pph 1448A and Pta 6605 is the same, as expected based on the analysis of gene clusters of the viosamine islands.The amino acid sequence of flagellin of Pto DC3000 (Group II) is slightly different from those of Group I bacteria such as Pta 6605 and Pph 1448A (Figure 5). The amino acid residue at position 183 is alanine in Pto DC3000 flagellin, and glycosylated serine is at that position in Pta 6605. However, the observed *m/z* values of the glycoprotein and glycopeptides (N136-R210) were 32,321 and 10,513, respectively, which were approximately 3,200∼3,300 larger than the calculated ones, respectively. The difference between observed and calculated values indicated the presence of six glycans composed of two rhamnoses and one modified viosamine residues (Table 2). The Asp-*N* digestion cleaved the peptide bond between A183 and D184 in Pto DC3000 flagellin, and the resultant glycopeptide (D168-A183) was shown by LC-ESI/MS to have two glycans (data not shown). In the amino acid sequence there was another Ser residue at 179 in addition to Ser176. Therefore, we concluded Pto DC3000 had six glycans, the structures of which seem to be identical to those of Pta 6605 at the 143, 164, 176, 179, 193, and 201 Ser residues.

**Table 2:** *m/z* values of intact flagellin and each peptide fragment *.

**pv.**	**intact (A2-Q282)** a			**N136-R210 b**			**D200-A211 b**		

	**calcd. [M+H] ^+^**	**Observed** c	**Δ**	**calcd. [M+H]^+^**	**Observed** c	**Δ**	**calcd. [M+H]^+^**	**Observed** d	**Δ**
Pta	29,147	32,487	3,300	7,388	10,611	3,220	1,276	1,813	537
Pph	29,147	32,545	3,400	7,388	10,616	3,230	1,276	e	
Pto	29,044	32,321	3,300	7,287	10,513	3,230	1,276	1,813	537
Psy	29,178	32,393	3,200	7,385	10,446	3,060	1,319	1,828	509
**pv.**	**D139-F167** b			**D168-A188** b			**E189-I199** b		

	**calcd. [M+2H]^2+^**	**Observed** d	**Δ**	**calcd. [M+2H]^2+^**	**Observed** d	**Δ**	**calcd. [M+H]^+^**	**Observed** d	**Δ**

Pta	1,440	1,977	537 × 2	954	1,491	537 × 2	1,107	1,644	537
Pph	1,440	e		954	e		1,107	e	
Pto	1,433	1,970	537 × 2	723	1,261	537 × 2	1,020	1,557	537
Psy	1,426	1,935	509 × 2	945	1,454	509 × 2	1,123	1,632	509

* The *m/z* values of A2-Q282 and N136-R210 are described using averaged atomic weights. Those of D139-F167, D168-A188, E189-I199 and D200-A21 are expressed as monoisotopic ions;

a There is no translation start codon (Met) in the mature flagellin;

b The digestion of flagellin by trypsin gave the glycopeptide N136-R210, and by Asp-*N* gave four glycopeptides, D139-F167, D168-A188, E189-I199 and D200-A211;

c Ions observed in the MALDI-TOF mass spectrum. The measurement errors for intact proteins and the trypsin-digested proteins are around 300 ppm and 100 ppm, respectively;

d Ions observed in ESI mass spectra;

e Not determined.

**Figure 7:**
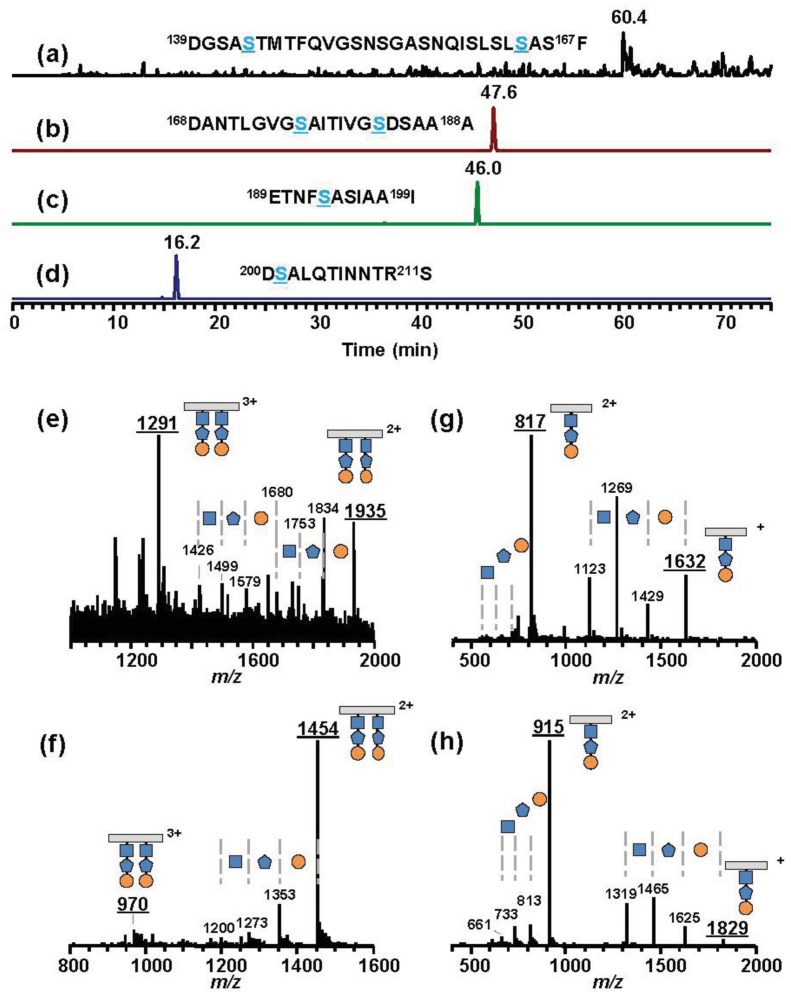
Extracted ion chromatograms (XIC) and mass spectra of *P. syringae* pv. *syringae* B728a. Asp-*N* digested glycopeptides were analyzed by LC-ESI/MS. XICs are of calculated *m/z* values in Table 2 corresponding to four glycopeptides, D139-F167 (**a**), D168-A188 (**b**), E189-I199 (**c**), and D200-S211 (**d**). Blue Ser residues have glycans. The dominant peak at 60.4 min in XIC (**a**) gave the mass spectrum (**e**), which showed [M+2H]^2+^ at *m/z* 1,935 ([3,869 (monoisotopic mass of glycosylated D139-F167) + 2 (two protons)]/2 (double charge)) and [M+3H]^3+^ at *m/z* 1,291 ([3,869 (monoisotopic mass of glycosylated D139-F167) + 3 (triple protons)] / 3 (triple charge)). The peak at *m/z* 1,834 is the fragment ion losing the terminal saccharide (orange circle). Other peaks at *m/z* 1,753, 1,680, 1,579, 1,499, and 1,426 are the fragment ions of a cascade of deglycosylation. The peaks in the XICs (**b**–**d**) also gave the mass spectra showing [M+H]^+^, [M+2H]^2+^, or [M+3H]^3+^ (the *m/z* values are underlined) accompanied by deglycosylated fragment ions (**f**–**h**, respectively). All glycopeptides have glycans composed of saccharides with masses of 203 (*N*-acetylated hexosamine, orange circle), 160 (methylated deoxyhexose, blue pentagon), and 146 (deoxyhexose, blue square) from the distal end. The symbol “+” or “2+” means the ion singly charged or doubly charged, respectively.

Psy B728a flagellin conserves six serine residues, which are glycosylated in Pta 6605, although the identity of the amino acid sequence is lower among pathovars of *P. syringae* (Figure 5). However, the molecular mass of flagellin glycopeptide (N136-R210) of Psy B728a (Group IV) was obviously smaller than those of other pathovars/strains (Figure 6 and Table 2). The molecular mass of one glycan is 509 (Table 2), indicating the glycan structure is different from those in Groups I and II and that there is no viosamine-related saccharide, as indicated by the analysis of the gene cluster of the vioamine island. Analysis of Asp-*N*-digested flagellin with LC-ESI/MS revealed that Psy 728a also had six glycans at the 143, 164, 176, 183, 193, and 201 Ser residues (Figure 7). The glycan was composed of saccharides with the mass values of 203, 160, and 146 from the non-reducing end, which corresponded to *N*-acetylhexosamine, methylated deoxyhexose, and deoxyhexose, respectively. In Pta 6605 and Pgl race 4, *fgt1* and *fgt2*, located upstream of *fliC*, encode flagellin glycosyltransferase, and Fgt1 transfers rhamnose to a serine residue [8]. Because all pathovars/strains of *P. syringae* investigated, including Psy 728a, conserve *fgt1* and *fgt2*, the proximal deoxyhexose of the glycan chain seems to be rhamnose. However, the detailed structural analyses regarding the kinds of deoxyhexose and hexosamine, the positions of modifications, and the linkage types between saccharides are under investigation.

## 3. Experimental Section

### 3.1. Bacteria and Culture Conditions

*Pseudomonas syringae* pv. *tabaci* 6605 WT and its mutant strains, pv. *phaseolicola* 1448A, pv. *tomato* DC3000, and pv. *syringae* B728a, were maintained in King's B (KB) medium at 27 °C, and *Escherichia coli* strains were grown at 37 °C in Luria-Bertani (LB) medium.

### 3.2. DNA Cloning and Generation of Mutant Strains

According to the genome sequence of *P. syringae* pv. *phaseolicola* 1448A (accession number, NC005773), a DNA fragment including the putative gene for methyltransferase (*vioM*, ortholog of PsPPH3421) was isolated by PCR using primer pairs M1 (5′-CTTTGCTGACGAAGTGACCG-3′) and M2 (5′-CACGACTTTGATGTCCGGGC-3 ') and genomic DNA from Pta 6605, and cloned into pGEM-T Easy plasmid vector. An internal region (201 bp) of *vioM* was deleted from the PCR-amplified DNA fragment by digestion with BsmI and subsequent self-ligation to construct a plasmid for mutant generation. To generate a mutant strain of putative 3-oxo-acyl-carrier protein reductase gene (*vioR*, ortholog of PsPPH3425), DNA fragments were amplified by PCR from genomic DNA of Pta 6605 using primer pairs R1 (5′-ATAATCCGGGCTTTGCGTGC-3′) and R2 (5′-cgcggatccATGCCAATAGATTGCTCATCG-3′) for the downstream of *vioR*, and R3 (5′-cgcggatccCCAACGTTCCTCTTCAGGGC-3′) and R4 (5′-TGTCAACCAGTTGCCGTTGC-3′) for the upstream of *vioR*, respectively (lowercase letters indicate an artificial sequence for BamHI digestion). Each amplified DNA fragment was ligated at the *Bam*HI site after *Bam*HI digestion, and the resulting fragments were amplified by PCR using the primer pair of R1 and R4 for the deletion of *vioR*. The *vioM*- or vioR-deleted DNA fragments were finally inserted into the mobilizable cloning vector *pK18mobSacB* [28] to generate pKdvioM and pKdvioR. Each resultant plasmid was introduced into *E. coli* S17-1 by electroporation and conjugated with Pta 6605 WT. After removal of the plasmid by incubation on KB agar plates containing 10% sucrose, each mutant strain was isolated.

### 3.3. Purification of Flagellin Proteins

Flagellin proteins were purified following the previously described procedure [29]. Briefly, each bacterium incubated in MMMF medium (50 mM potassium phosphate buffer, 7.6 mM (NH_4_)_2_SO_4_, 1.7 mM MgCl_2_, and 1.7 mM NaCl, pH 5.7, supplemented with 10 mM each of mannitol and fructose) was harvested by centrifugation and then resuspended in 50 mM sodium phosphate buffer (pH 7.0). The flagella were sheared off by vortexing and then separated by centrifugation. Flagella in the supernatant were filtered through a 0.45-μm pore filter for sterilization. The filtrate was further centrifuged at 40,000 rpm for 30 min at 4 °C, and the resultant pellet was suspended in sterile H_2_O.

### 3.4. Preparation of Glycosylated Peptides

The purified flagellins were digested with trypsin (proteomics grade, Sigma-Aldrich, MA, USA) or Asp-*N* endopeptidase (Takara Bio, Shiga, Japan) at 37 °C for 18 h in 10 mM Tris-HCl buffer (pH 8.0). The obtained peptide mixture was directly subjected to mass analysis.

### 3.5. MALDI-TOF Mass Spectrometry

MALDI-TOF mass spectra were recorded on a Reflex II (Bruker Daltonik GmbH, Bremen, Germany) or a 4800 MALDI TOF/TOF analyzer (AB SCIEX, CA, USA) using matrices of α-cyano-4-hydroxycinnamic acid (Bruker Daltonics, Inc., MA, USA), 2, 5-dihydroxybenzoic acid (Bruker Daltonics), or sinapinic acid (Bruker Daltonics) in the positive-ion mode with an acceleration voltage of 20 kV. Both mass spectrometers were tuned and calibrated using commercially available standard proteins (Bruker Daltonics; Protein calibration standard I containing insulin, ubiquitin, cytochrome C, and myoglobin or Protein calibration standard II containing trypsinogen, Protein A, bovine albumin) prior to measurements or during the measurements.

### 3.6. LC-ESI Mass Spectrometry

LC-MS/MS experiments were performed on an LCQ mass spectrometer (Thermo Fischer Scientific, CA, USA). Peptide mixtures obtained by treatment with Asp-N were applied to a semimicro-LC system (SI-2, Shiseido, Tokyo, Japan) and separated into glycosylated peptides on a C8 column (Zorbax 300SB-C8, 2.1 × 150 mm, 5 μm, Agilent Technology, CA, USA). The proportion of solvent B (80% acetonitrile containing 0.1% formic acid) to solvent A (0.1% formic acid) was linearly increased from 6% to 36% over 80 min at a flow rate of 200 μL/min at 40 °C. The eluate was introduced into the mass spectrometer, which was preliminarily tuned with a solution of a commercially available peptide, substance P, in the positive-ion mode under the following conditions: ESI spray voltage, 4.5 kV; capillary temperature, 210 °C; capillary voltage, 46 V; sheath gas (nitrogen) flow rate, 60 (arbitrary units); auxiliary gas flow rate, 20 (arbitrary units). Mass spectra were recorded over the mass range of *m/z* 350-2000.

## 4. Conclusions

In this study we identified the genes involved in the biosynthesis of dTDP-mVio as a viosamine island in Pta 6605, and we generated mutant strains for individual genes of the viosamine island. Molecular masses of flagellin glycopeptides from the mutant strains and the predicted enzymatic function of each gene product revealed each step of the biosynthetic pathway for dTDP-mVio. Viosamine islands in 8 pathovars and altogether 16 strains can be classified into four groups. Group I contains five pathovars and eight strains. The flagellin glycan of pv. *phaseolicola* 1448A was characterized as a representative strain of Group I, and was identical to that of Pta 6605. All strains of pv. *tomato* belong to Group II, and the flagellin glycan of representative strain DC3000 was found to be of the mVio-Rha-Rha type. However, Group III, pv. *syringae* 642, conserves only *vioA, vioM*, and *vioT*, and the structure of flagellin glycan is not clear. The flagellin glycan of pv. *syringae* B728a was investigated as a representative strain of Group IV and was found to have a different structure containing deoxyhexose, methylated deoxyhexose, and *N*-acetylhexosamine. These results indicate the universality of flagellin glycosylation and also the diversity of flagellin structure.
